# PEComa progressed by mTOR inhibitor therapy with anti-VEGFR TKI combined with PD-1 inhibitor: a case report

**DOI:** 10.3389/fonc.2025.1628650

**Published:** 2025-09-08

**Authors:** Guannan Wang, Lili Sun, Jian Guan, Boyan Yang

**Affiliations:** ^1^ National Cancer Center/National Clinical Research Center for Cancer/Cancer Hospital & Shenzhen Hospital, Chinese Academy of Medical Sciences and Peking Union Medical College, Department of Integrative Oncology, Shenzhen, China; ^2^ National Cancer Center/National Clinical Research Center for Cancer/Cancer Hospital & Shenzhen Hospital, Chinese Academy of Medical Sciences and Peking Union Medical College, Department of Pathology, Shenzhen, China

**Keywords:** malignant perivascular epithelioid cell tumor, TSC2 mutation, mTOR inhibitor, anti-VEGFR TKI, PD-1 inhibitor

## Abstract

Malignant perivascular epithelioid cell tumors (PEComas) are rare tumors in which the PI3K/AKT/mTOR pathway is critical for pathogenesis and is often associated with mutations in the TSC1/TSC2 genes. First-line mTOR inhibitors are an effective treatment, but metastatic PEComas eventually develop drug resistance and disease progression. A 76-year-old woman presenting with a painful soft tissue mass in the buttocks was diagnosed with malignant PEComa with local invasion and multiple distant metastases, and the tumor cells harbored a TSC2 gene mutation. After disease progression by mTOR inhibitor treatment, immunotherapy combined with anti-angiogenic therapy was enabled, which enabled the patient to achieve a 3-year survival benefit and provided a reference for the treatment of patients with refractory malignant PEComa.

## 1 Introduction

Perivascular epithelioid cell tumor (PEComa) is a rare tumor of mesenchymal origin that expresses differentiation features of melanocytes and smooth muscle cells (1). Folpe et al. (2) classified PEComas as benign, potentially malignant, and malignant based on tumor diameter, nuclear grading, cytoarchitecture, mitotic rate, degree of necrosis, vascular invasion, and infiltrative growth. No clear diagnostic criteria have been established for malignant PEComas. According to statistics, the global incidence of malignant PEComa is 0.12-0.24 per million people (1), it can occur at any age, with a median age of 39–54 years, and is more common in women, with a male-to-female ratio of about 1:2.5. Distant metastases are found in approximately 10-30% of PEComa patients at the time of initial diagnosis, and the lung is the most common site of metastasis with an incidence of 90%, followed by the kidney and liver. Malignant PEComa is insensitive to standard chemotherapy and radiotherapy, and radical surgical resection is the key to cure. In recent years, molecular detection studies have revealed that malignant PEComa is often accompanied by mutational inactivation of TSC1 and/or TSC2 genes, leading to activation of the mammalian target of rapamycin (mTOR) pathway (3) and partially carries TFE3 gene fusion. Positive efficacy has been achieved by applying mTOR inhibitors for the treatment of PEComa (4–8). mTOR inhibitor-resistant backline therapy currently has no recommended standard regimen. In our report, we present a case of a metastatic PEComa patient who progressed on mTOR inhibitor therapy with clinical benefit after treatment with VEGFR-TKI in combination with a PD-1 inhibitor, with a disease control time of 13 months.

## 2 Case report

**Figure 1:**
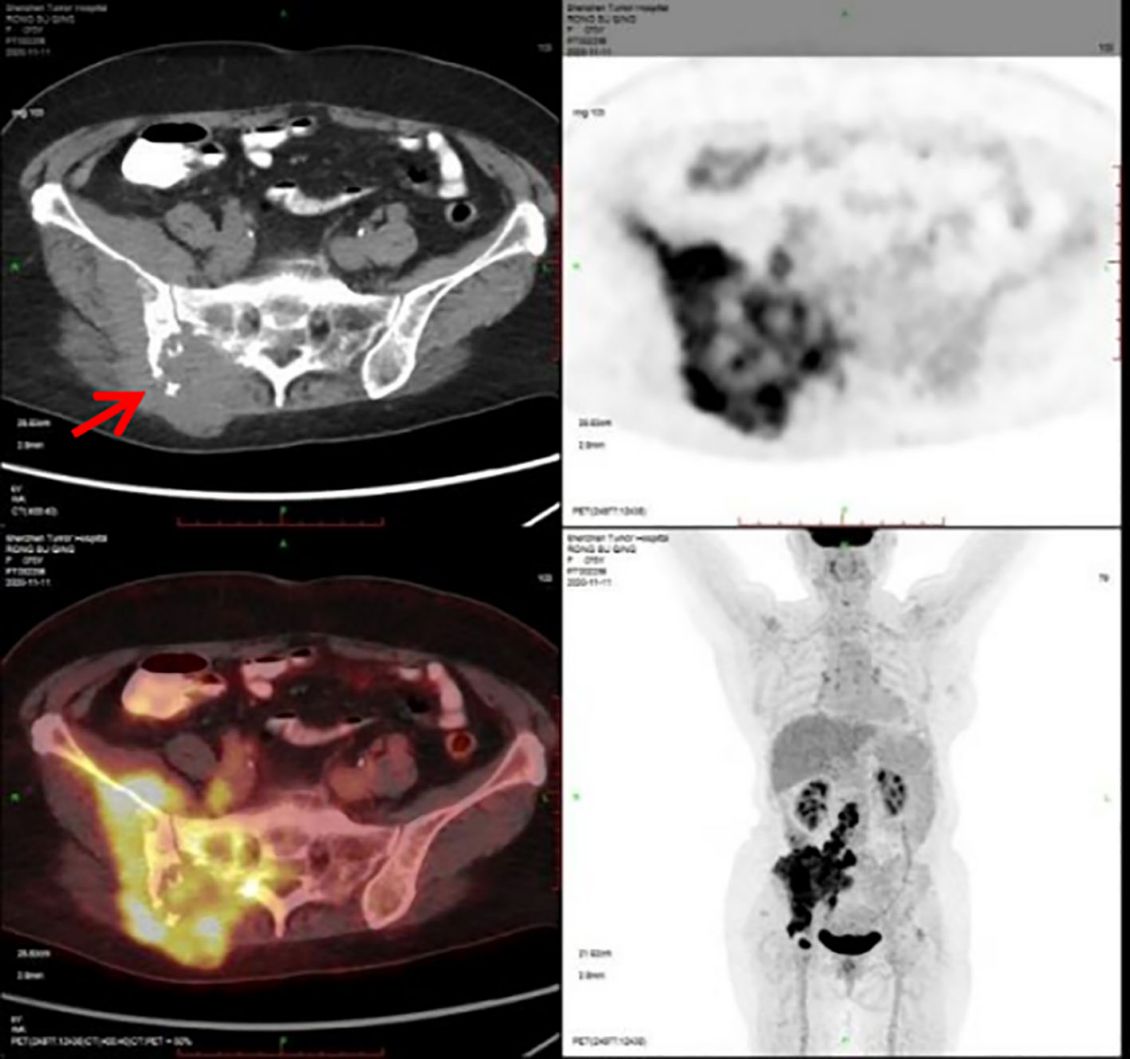
PET-CT scan.

A 76-year-old woman presented with six-month history of gradually increasing right buttock mass with pain radiating to the right lower limb. She denied any anomalies in the past and in the family history. She had a past history of coronary artery disease, hypertension and post coronary stenting. Physical examination revealed a hard and fixed mass measuring 5.0 cm×4.0 cm on the right buttock and a 1.5cm×1.0cm enlarged lymph node in the right groin. The Eastern Cooperative Oncology Group (ECOG) score was 2 points. The Numerical Rating Scale (NRS) score was 8 points. The 11^th^ November of 2020 the Positron Emission Tomography/Computer Tomography (PET-CT) scan showed bone destruction of the right ilium and sacrum and soft tissue mass in the right gluteus maximus, iliacus and erector spinae with a maximal cross-section of 11.8cmsectio, exhibiting increased radioactive uptake with a maximum standardized uptake value (SUVmax) of 10.3. Multiple nodules on the right psoas major, psoas rectus and right gluteus measuring largest 2.9cm×1.6cm exhibit increased radioactive uptake with a SUVmax of 13.5. Lumbar 3 and 5 vertebral spinous processes exhibit increased radioactive uptake with a SUVmax of 9.9. Multiple enlarged lymph nodes in retroperitoneum, right iliac paravascular and inguinal area exhibit increased radioactive uptake with a SUVmax of 12.2, with the largest lymph node size of 4.0cm×3.1cm. Multiple nodules in both lungs exhibit mild increased radioactive uptake with the SUV values of 1.3 and 1.8 before and after delayed scanning (**Figure 1**).

**Figure 2:**
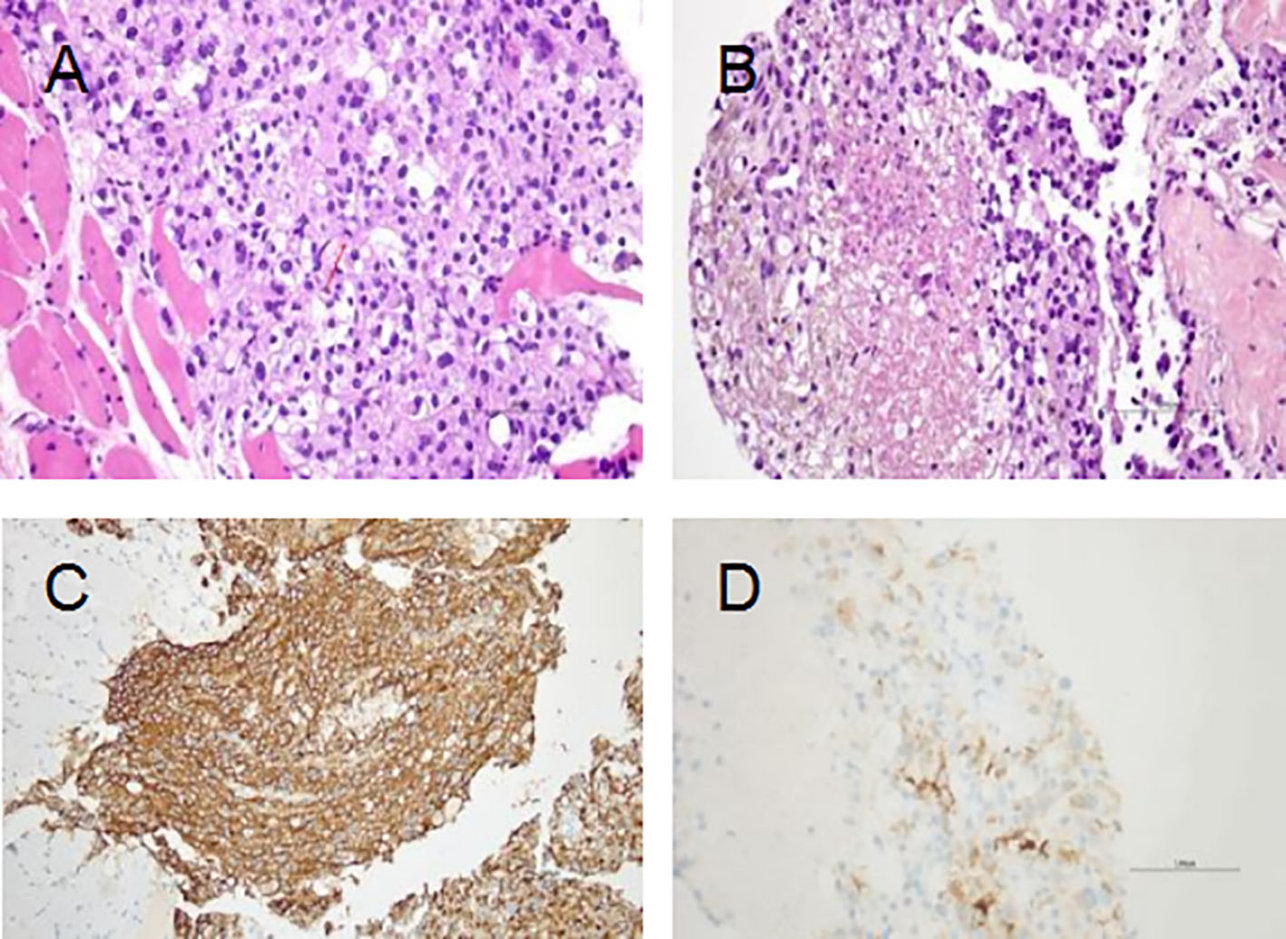
Hematoxylin and eosin (H&E) staining and immunohistochemical analyses. **(A)** The tumor grows infiltratively in the rhabdomyosarcoma tissue; the tumor cells are cytoplasmic and have a "spider cell" morphology, and some of them have distinct nuclei; the red arrow indicates nuclear schizophrenic image (×400). **(B)** Tumor necrosis (×400). **(C)** HMB-45 (strong and diffuse). **(D)** Melan-A (Focal).

**Figure 3:**
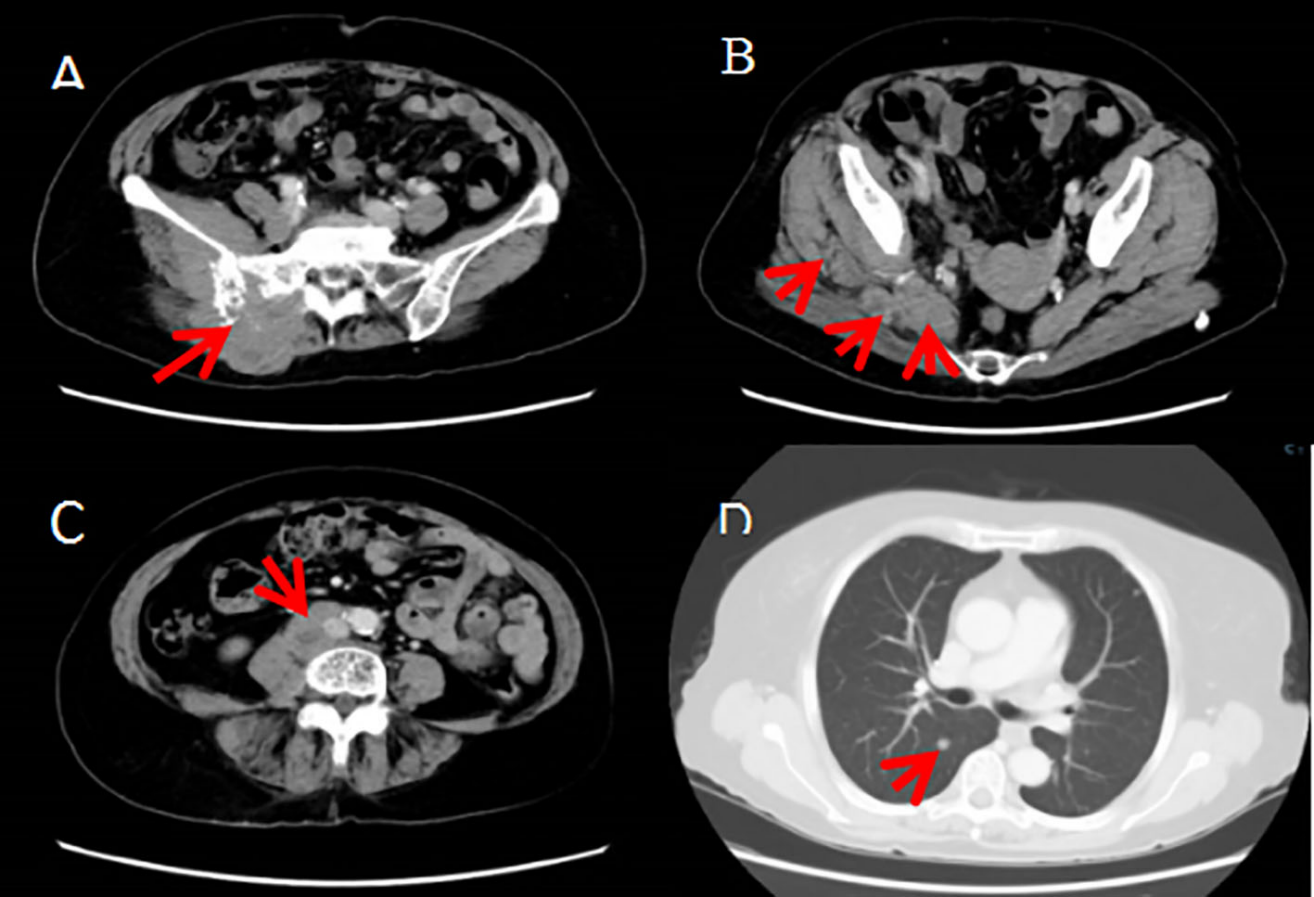
**(A–C)**The right buttock tumor invaded the right ilium, sacrum and sacral nerve, and involved the right erector spinae, iliopsoas, gluteus and pyriformis muscles and the tumor metastasized to the right psoas major and psoas square muscles as well as the retroperitoneum, right iliac paravascular, right inguinal lymph nodes. **(D)** The tumor metastasized to both lungs.

The patient underwent a puncture biopsy of the right buttock mass on December 18, 2020. The pathology results suggested a malignant tumor of mesenchymal origin with large necrotic areas. The tumor tissue grew infiltratively in the transverse muscle and fibro-adipose tissue, and the tumor tissue was arranged in the form of nests and sheets, and the central cytoplasm of the tumor cells was eosinophilic red, and the cytoplasm around the periphery was hyaline, which was the so-called spider cells, and the nuclei of some of the tumor cells were distinctive; the nuclei were seen to be split into 2–3 nuclei/50 HPF and necrotic. Immunohistochemical staining results showed: AE1/AE3 (–), Desmin (–), HMB-45 (3+), INI1 (+), Ki-67 (hotspot +20%), Melan-A (1+), MyoD1 (–), S-100 (–), SMA (–), TFE3 (3+), Vimentin (2+) (**Figure 2**). The pathology results confirmed the diagnosis of malignant perivascular epithelioid cell tumor (PEComa) in right buttock. Meanwhile, enhanced CT scans were performed to further define the site of tumor invasion and metastasis (**Figure 3**). Genetic testing of tissue specimens revealed a TSC2 gene mutation (p.N398Tfs*27 Exon12) with a mutation abundance of 24.21%. Tumor Proportion Score (TPS) and Immune Proportion Score (IPS) of Programmed Cell Death 1 Ligand 1 (PD-L1) was 10% and <1%. Tumor Mutation Burden (TMB) was 0.56 Muts/Mb. And the tumor was Microsatellite Stability (MSS).

**Figure 4:**
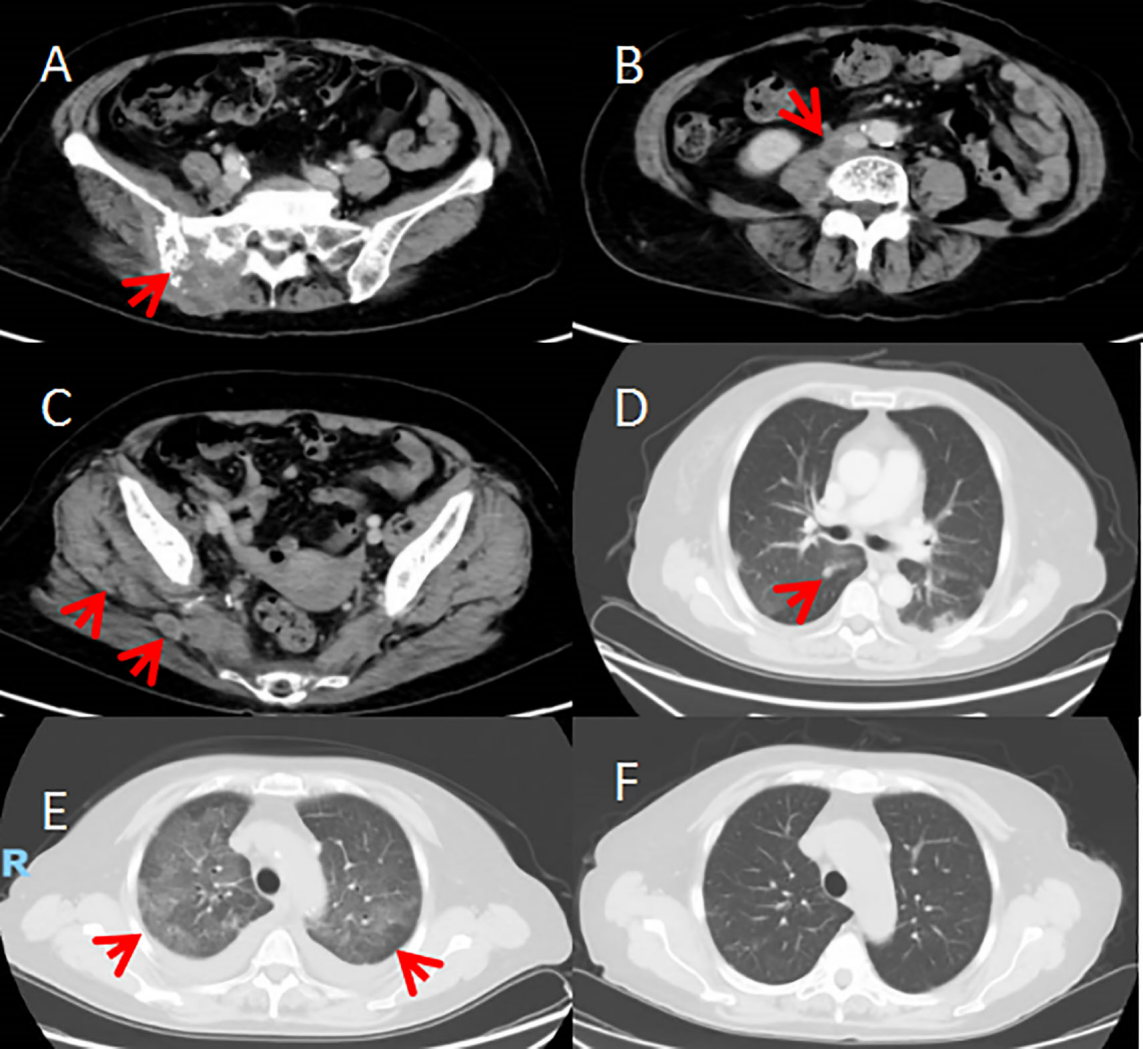
**(A–D)** After 1 month of treatment on February 1, 2021. CT scan showed the tumour was smaller than before; multiple flaky ground-glass shadows in both lungs (grade 1 interstitial pneumonia). **(E)** CT scan on March 4th showed diffuse multiple flaky ground-glass density shadows in both lungs (grade 2 interstitial pneumonia). **(F)** After treatment, CT scan on March 26th. showed the diffuse multiple flaky ground-glass density shadows in both lungs were significantly better than before (grade 1 interstitial pneumonia).

Due to the patient’s extensive tumor invasion, local treatments such as surgery, radiation therapy, and interventional therapy were not appropriate. On January 1 of 2021 the patient was started on everolimus 10mg once daily for about 1 week, the patient successively experienced adverse reactions (graded according to the NCI CTCAE criteria for adverse events): elevated blood glucose, oral mucositis, urinary tract infection, neurovascular edema, grade 1 elevated liver enzymes, grade 1 interstitial pneumonitis, and the dose of everolimus was reduced to 5mg once a day, which was tolerable. The patient’s pain symptoms gradually improved, and CT scan on February 1,2021 showed 26% tumor shrinkage (**Figures 4A–D**), and the efficacy was assessed as stable disease(SD) according to the solid tumor efficacy evaluation criteria RECIST 1.1. And then everolimus was suspended from March 3 to March 29 because of the development of grade 2 interstitial pneumonia (**Figure 4E**). The patient improved with glucocorticoid and anti-infective therapy (**Figure 4F**) and continued to take everolimus. Repeat CT scans on June 1 of 2021 suggested 29% tumor shrinkage. In order to enhance the efficacy, start everolimus combined with anlotinib 8mg once a day, consecutive 14 days and stopping 7 days, repeated every 3 weeks. Because the patient occurred in the inferior vena cava and the right common iliac vein thrombosis in April 21, 2022, discontinued the use of anlotinib, continue to single-drug everolimus treatment. Disease stabilization with regular follow-up. The progression-free survival was 17 months.

On June 7, 2022, the patient’s symptoms of chest tightness and shortness of breath worsened, and chest CT scan showed new pleural metastasis, pleural effusion, and essentially the same changes of interstitial pneumonia in both lungs. Cytopathology of the pleural fluid showed malignant tumor cells, consistent with PEcoma metastasis. CT scan showed that the local involvement of the tumor increased, multiple metastases increased, multiple lymph node metastases increased, and involved the right internal iliac artery, right ureteral abdominal segment with the right pelvic calyx, ureteral dilatation, and the right kidney was hydronephrotic. The patient’s serum creatinine increased to 110umol/L (normal range 45-84umol/L) and right ureteral stenting was performed to relieve the right hydronephrosis caused by tumor compression, and then the serum creatinine returned to normal. On June 23, 2022, the second puncture biopsy pathology of the right buttock tumor was performed and malignant PEComa was considered in conjunction with immunohistochemistry, Ki-67 (hotspot +25%), TFE3 (–).Genetic testing was performed again and showed no TSC 1/2 gene mutation, MSS and TMB 1.0 Muts/Mb.

On August 17, 2022, the treatment regimen was changed to anlotinib combined with toripalimab. Denosumab 120mg subcutaneously, repeated every 28 days. This treatment regimen was effective for 11 months and the patient had improved physical status, an ECOG score of 1, a significant reduction in pain, an NRS score of 0, creatinine in the normal range, no significant discomfort and tolerable side effects. The progression-free survival was 11 months.

On 01 August 2023, the patient’s cough and wheezing worsened and CT showed bilateral diffuse pleural metastases, bilateral diffuse multiple metastases in both lungs worsened from before and progressed. The treatment regimen was changed to sorafenib in combination with toripalimab. Pneumothorax, lung infection and increased pleural effusion appeared on 15 November 2023, and CT on 30 November 2023 suggested increased and enlarged diffuse metastases in both lungs and worsening of carcinomatous lymphadenitis compared with the previous period, and the patient developed respiratory failure and died clinically on 24 December 2023. The patient’s overall survival time was 3 years.

## 3 Discussion

### 3.1 Epidemiology

The pathogenesis of PEComa is unclear, and because its cells express neurogenic and myogenic markers, it is suggested that it may be derived from pluripotent stem cells that can differentiate into multiple cell types. Bonetti first introduced the concept of perivascular epithelioid cells (PEC) in 1992 and suggested that clear cell carcinoma of the lung and vascular smooth muscle lipoma of the kidney belong to the same tumor family (9). However, PEC cells are not found in normal humans, and the tissue origin and pathogenesis of PEComas remain unclear. There is a clear female predominance in the gender of patients, especially in women of reproductive age. Expression of ER and/or PR has been found in some tumors and it is speculated that their occurrence is hormone-related.

The clinical presentation of PEComa is complex and morphologically diverse, with tumor cells consisting of large numbers of round and ovoid perivascular epithelioid cells expressing melanoma markers and pigment-specific antibodies. PEComa is common in middle-aged women and often occurs in the uterus, gastrointestinal tract, genitourinary system and retroperitoneum, and it is rare in soft tissue, bone and skin (10). The diagnosis of PEComa is based on histopathology and immunophenotype, supplemented by molecular pathology when necessary. Genetic studies suggest that it is associated with mutations in the tumor suppressor genes TSC1 or TSC2. The protein complex encoded by TSC1/TSC2 negatively regulates the mTOR signaling pathway, which is involved in the regulation of a number of biological processes including cell growth, proliferation, metabolism, transcription and autophagy (3).

### 3.2 Pathological feature

Most PEComa are benign tumors, and a few cases have malignant biological behavior with distant metastasis. There is no clear consensus on the criteria for diagnosis of malignancy, Folpe et al (2) summarized 26 cases of PEComa of soft tissue and genital system, and indicators suggesting malignant biological behavior. This case of perivascular epithelial tumor arising in the soft tissue reported in this paper reached a maximum diameter of 12.4 cm and showed infiltrative growth in the soft tissue of the buttocks, with visible necrosis and readily visible nuclear schizophrenic images, which meets the diagnostic criteria for malignant PEComa. In addition, multiple systemic metastases were found at the same time, which was consistent with the clinical features of malignant tumor.

The immunophenotype of the tumor in this case also supported the diagnosis of a perivascular cell tumor, along with the expression of HMB45 and MelanA and the absence of S-100 expression, which excluded the diagnosis of malignant melanoma. It is worth noting that the lesion in this case had strong positive expression of TFE3, which overlapped with the immunophenotype of adenovascular soft tissue sarcoma, and also in terms of site of occurrence and pathomorphology, which in some cases needs to be differentiated from adenovascular soft tissue sarcoma, but adenovascular soft tissue sarcoma does not express pigmented cellular markers, and therefore the diagnosis of adenovascular soft tissue sarcoma is not supported in this case. Rearrangement or amplification of the TFE3 gene has been reported in approximately 30% of PEComas in the literature (11), so immunohistochemical examination may detect positive TFE3 protein expression.

PEComa’s cellular origin remains unclear, but it exhibits both melanocytic (HMB45, MelanA, MiTF, Cathepsin K) and myogenic (SMA, desmin) differentiation markers. While HMB45 shows high sensitivity (92-100%), it can be negative in rare cases (2, 12); Cathepsin K demonstrates exceptional sensitivity (93-100%) and is often retained in HMB45-negative tumors. Heterogeneity in marker expression is observed, with approximately 9% of cases reported to be negative for Melan-A but positive for MiTF. Some studies have suggested that cases losing melanocytic markers may have a poorer prognosis, potentially reflecting tumor dedifferentiation (13), and warrant closer follow-up. However, these conclusions should be interpreted with caution due to the currently limited sample sizes.

In addition, genetic testing in this case revealed mutations in the TSC gene, which supports the diagnosis of PEComa. TSC1 and TSC2 encode hamartin and tuberin proteins, and mutations lead to tumorigenesis after inactivation of these tumor suppressor genes. Mutations in the TSC1/TSC2 genes lead to activation of the mammalian target of rapamycin (mTOR) signaling pathway, which is the molecular basis for the clinical application of mTOR pathway inhibitors for the treatment of PEComa. 1/3 to 1/2 of PEComa is clinically associated with tuberous sclerosis complex (TSC) (11) with germline mutations in TSC1 (9q3.4) and TSC2 (16p13.3) genes, this case did not detect germline mutations in the TSC gene and is consistent with a case of sporadic PEComa.

In terms of molecular characterization, common gene mutations in PEComa included TP53 (47%), ATRX (32%) and MSH3 (17%), in addition to 11% and 29% mutation rates in TSC1 and TSC2 genes, respectively, and the others were fusion of transcription factor E3 (TFE3), rearrangement of RAD51 B gene (14). The incidence of TP53 mutations was lower in tumors with TSC1/2 mutations compared to TSC1 wild-type tumors (25% vs. 60%, p = 0.055). Mutations in MSH3 (25%) and ERCC2 (14%) were only reported in tumors with TSC1/2 mutations (3). TSC2 mutant tumors have a hyperactivated PIK3-Akt-mTOR pathway, and TFE3 translocation mutations activate MET signaling through transcriptional upregulation. Although TSC1/2 and TFE3 recombination are thought to be mutually exclusive, it has been reported that TSC1/2-mTOR pathway and TFE3 overexpression may be detected simultaneously (14). PEComas are also characterized by specific transcriptomic features of the overexpressed DAPL1, MLANA, SULT1C2, GPR143 and CHI3L1 genes, as well as epigenetic features of lysosomal and melanocyte proteins as biomarkers (15). In addition, the PEComa tumor microenvironment is characterized by a significant increase in the number of NK and fibroblasts, and a decrease in the number of CD8+ T cells and B cells (3). There is no significant difference in PFS or OS when TSC1 and TSC2 mutations are treated with mTOR inhibitors (16).

### 3.3 Treatment

Complete surgical resection with negative margins is the foundation for a cure. Since tumors are relatively resistant to chemotherapy and radiotherapy, there is no evidence that adjuvant chemotherapy or radiotherapy is beneficial. Molecularly targeted therapies, guided by the molecular characterization of tumors, are currently the mainstay of systemic therapy (17).

#### 3.3.1 Surgery, radiotherapy and chemotherapy

Malignant PEComa is insensitive to standard chemotherapy and radiotherapy and radical surgical resection is the key to cure (18). The efficacy of adjuvant or neoadjuvant radiotherapy in PEComa has only been reported in case reports and has not been clinically proven. Due to the importance of surgical treatment for long-term survival, patients with initially diagnosed advanced or metastatic PEComa have a poor prognosis. However, due to the limited response to chemotherapy and radiotherapy, there is a lack of data on cytoreductive surgery with non-R0 resection for advanced PEComa in the presence of potentially resectable and resectable metastases, with reference to other sarcomas, the likelihood as well as the risks and benefits of surgery need to be assessed, and some patients may benefit from neoadjuvant therapy. MDT discussions should be held before treatment and clinical trials are encouraged.

Commonly used chemotherapeutic agents for malignant PEComa include gemcitabine, docetaxel, adriamycin, isocyclophosphamide. Wagner (6) et al. reported partial remission of gemcitabine in combination with docetaxel in patients with metastatic PEComa. Adriamycin and isocyclophosphamide are standard chemotherapeutic agents for soft tissue sarcoma, but have limited efficacy in malignant PEComa.

#### 3.3.2 m-TOR inhibitors

Longer-term therapeutic responses with significantly improved efficacy over chemotherapy can be achieved with mTOR inhibitors in malignant PEcoma, especially in patients with mutations in the TSC1/TSC2 genes that result in aberrant activation of the mTOR pathway. mTOR inhibitors block cell proliferation, metabolism and angiogenesis signaling pathways by inhibiting the activity of the mTORC1 complex. mTOR inhibitors include everolimus, sirolimus, temsirolimus, and others. Albumin-bound sirolimus is a novel mTOR inhibitor that improves drug delivery efficiency and tumor targeting via an albumin carrier. The AMPECT trial (6) evaluated the significant anti-tumor activity of nab-sirolimus in patients with advanced or metastatic malignant PEComa carrying mutations in the TSC1/TSC2 genes or activation of the mTOR pathway. An objective remission rate (ORR) of 39% was achieved, with 2 patients achieving complete remission after long-term follow-up, 58% of patients sustained remission for more than 2 years, and the median progression-free survival (PFS) was 10.6 months and the median duration of response was 39.7 months. There was durable disease control and survival. Accordingly in November 2021 the US Food and Drug Administration (FDA) approved the marketing of Fyarro (Albumin-bound sirolimus) for the treatment of locally advanced unresectable or metastatic, which is the first FDA-approved drug for the treatment of advanced malignant PEComa in adults and is now considered a standard first-line treatment option. Patients with PEComa harboring TSC2 mutations who are treated with mTOR inhibitors have a better objective response rate and progression-free survival than patients with TSC1 mutations. Other mTOR inhibitors, including everolimus, temsirolimus, and sirolimus, have shown similar efficacy in retrospective studies.

#### 3.3.3 Targeted TFE3 translocation

Recently, a class of PEComa subtypes carrying TFE3 gene rearrangements has been identified (19). About 50 cases have been reported to date. These TFE3-rearranged PEComas predominantly consist of alveolar and epithelioid cells with low immunoreactivity for muscle markers and high immunoreactivity for TFE3 (3+) (20). Patients tend to be younger and have no association with tuberous sclerosis. TFE3-translocated PEComas are less responsive to mTOR inhibitors (21), and the low response rate is associated with MET pathway activation, which is one of the mechanisms of mTOR inhibitor resistance. However, there is a lack of clinical studies evaluating mTOR inhibitors in PEComas with or without TFE3 translocation. TFE3 fusion has been found to activate MET signaling through transcriptional upregulation. Therefore, c-MET inhibitors may be more effective in TFE3-altered PEComas (18). Therefore, it has been proposed that PEComas can be subdivided into two molecular subgroups: type 1, which is responsive to mTOR inhibitors, and type 2, which is responsive to c-MET inhibitors. However, there is still a lack of supporting data. While it is generally accepted that TFE3 rearrangements and TSC1/2 mutations are mutually exclusive, our PEComa patient had a TSC2 mutation and TFE3 phenotypic alteration.

#### 3.3.4 Anti-angiogenesis inhibitors

Anti-angiogenic therapy blocks the process of tumor angiogenesis by inhibiting the activity of tumor angiogenic factors, thereby cutting off the nutrient supply to the tumor and inhibiting tumor growth and metastasis. The main targets include vascular endothelial growth factor (VEGF), platelet-derived growth factor (PDGF) and fibroblast growth factor (FGF). In the VEGF/VEGFR pathway VEGF-A is a core regulator of angiogenesis and promotes endothelial cell proliferation and angiogenesis through the VEGFR-2 signaling pathway. Small molecule tyrosinase inhibitors such as Sorafenib, Sunitinib and Pazopanib are multi-targeted inhibitors that exert anti-angiogenic effects by inhibiting VEGFR, PDGFR and other pathways, and have achieved partial remission in several PEComa patients (22, 23). The therapeutic efficacy of single-agent angiogenesis inhibitors for advanced PEcoma is often limited, and the therapeutic strategy of VEGFR inhibitors combined with mTOR inhibitors exerts a synergistic effect, blocking the survival and proliferation signals of tumor cells from different angles and reducing the possibility of tumor cells to develop drug resistance through activation of other bypass signaling pathways, thus delaying the emergence of drug resistance (24). The incidence of ILD caused by m-TOR inhibitors has been reported to be approximately 0.4% to 10%, and the mechanism may involve direct cytotoxicity, immune-mediated inflammatory response, and vascular endothelial damage (25, 26). In this case, the patient was discontinued after everolimus therapy due to intolerable interstitial pneumonitis, and the disease progressed after restarting therapy at a reduced dose. Subsequent combination of antiangiogenic therapy with VEGFR inhibitors (amlotinib) maintained disease stabilization for 10 months without further exacerbation of interstitial pneumonitis. There is no conclusive evidence that VEGFR inhibitors may have an effect on m-TOR inhibitor-induced interstitial lung disease. VEGFR inhibitors may attenuate m-TOR inhibitor-induced lung injury by inhibiting angiogenesis and inflammatory responses in an animal model of pulmonary fibrosis. Some clinical studies have also reported the effectiveness of nidanib, a VEGFR inhibitor, in slowing the progression of systemic sclerosis-associated interstitial lung disease (SSc-ILD) (27).

#### 3.3.5 Immune checkpoint inhibitors

Malignant PEComa is a rare and aggressive subtype of soft tissue sarcoma with a low response rate to conventional chemotherapy, while immune checkpoint inhibitors (ICIs) have received attention for their potential efficacy in tumors with high tumor mutation load (TMB) or high PD-L1 expression. There are case reports of objective remission rates (ORR) of 17%-50% for ICIs alone in malignant PEComa with high TMB-H or PD-L1 expression (28, 29). In patients carrying patients carrying TSC1/2 mutations everolimus combined with pembrolizumab synergistically inhibits tumor growth and enhances immune response. Theoretically, ICIs combined with anti-angiogenic drugs can improve the tumor microenvironment and enhance the efficacy of ICIs through “vascular normalization”. In this case, the patient obtained 11-month disease control after multiple lines of therapy with amlotinib in combination with teraplizumab. Local radiotherapy or selective internal radiation therapy (SIRT) can enhance the immune-activating effect of ICIs and provide new ideas for combination therapy. Currently, there are relatively few cases of immunotherapy application in malignant PEcoma, and more clinical trials and basic research are still needed to further clarify the predictive markers of immunotherapy efficacy, explore the optimal combination therapy regimen, and thoroughly study the resistance mechanism of immunotherapy, in order to improve the therapeutic efficacy of malignant PEcoma and improve the prognosis of patients.

#### 3.3.6 Combination therapy

Although the current standard first-line drug, albumin-conjugated sirolimus, improves the efficacy of PEComa significantly, it is not satisfactory. Combination therapy may save more patients.Anti-PD-1 immunotherapy combined with radiotherapy benefits patients who are not eligible for radical resection, providing relief from cancer-related pain and bleeding. Sanfilippo et al. (30) evaluated the efficacy of adding anti-estrogen therapy to the treatment of female patients who were resistant to mTOR inhibitors. The study found an ORR of 43% and a DCR of 86%. Combination anti-angiogenic agents, such as pazopanib and sunitinib, are also under study (31). A targeted or immune combination with gemcitabine and/or albumin nanopaclitaxel-based chemotherapy could also be a second-line option (18). Known mechanisms of resistance to mTOR inhibitors from studies of other tumors include mutations in the structural domains of TSC1/TSC2 kinases; activation of PI3K/AKT or MAPK/ERK pathways; overexpression of ABC transporter proteins; activation of METs; and changes in the microenvironment. Future strategies to overcome resistance, such as using dual PI3K/AKT inhibitors or C-MET inhibitors in combination with anti-vascular therapies, deserve further investigation (32).

### 3.4 Strengths and limitations

This case represents a rare instance of refractory malignant PEComa, a condition that required the administration of multiple lines of combination therapy, resulting in a survival benefit. This case offers a valuable reference point for similar cases encountered in clinical practice. However, given the inherent variability among individual cases, further validation through clinical trials is necessary to ascertain the efficacy and safety of combination therapy involving anti-angiogenic therapy and immune checkpoint inhibitors as a backline treatment. Furthermore, the case was negative for TSC mutation subsequent to conventional treatment for disease progression, which may be attributable to tumor heterogeneity or the sensitivity of the genetic testing technology on the one hand, and the genetic alteration of the tumor after treatment on the other hand.

Malignant PEComa is a relatively rare tumor type that is insensitive to conventional chemotherapy and radiotherapy, and there is a lack of standard treatment options. In clinical practice, treatment is mostly individualized according to the patient’s specific situation. Therefore, finding new therapeutic strategies is particularly important. With the in-depth study of the molecular biology of PEComa, molecular targeted therapy has gradually become a new direction in the treatment of PEComa, and the combination of two important molecularly targeted drugs, VEGFR inhibitors and immune checkpoint inhibitors, has a potential application in the treatment of PEComa. Targeted therapy occupies an important position in the treatment of advanced PEComa, but still faces the risk of post-treatment resistance or disease progression, so the combination therapy model needs to be explored.

## Data Availability

The raw data supporting the conclusions of this article will be made available by the authors, without undue reservation.
